# Adaptable Invisibility Management Using Kirigami-Inspired Transformable Metamaterials

**DOI:** 10.34133/2021/9806789

**Published:** 2021-09-10

**Authors:** He-Xiu Xu, Mingzhao Wang, Guangwei Hu, Shaojie Wang, Yanzhao Wang, Chaohui Wang, Yixuan Zeng, Jiafang Li, Shuang Zhang, Wei Huang

**Affiliations:** ^1^Air and Missile Defense College, Air Force Engineering University, Xi'an 710051, China; ^2^Institute of Flexible Electronics, Northwestern Polytechnical University, Xi'an 710072, China; ^3^Department of Electrical and Computer Engineering, National University of Singapore, Singapore, Singapore 117583; ^4^Centre for Quantum Physics, Key Laboratory of Advanced Optoelectronic Quantum Architecture and Measurement (MOE), Beijing 100081, China; ^5^School of Physics, Beijing Institute of Technology, Beijing 100081, China; ^6^Department of Physics, University of Hong Kong, Hong Kong, China; ^7^Department of Electronic & Electrical Engineering, University of Hong Kong, Hong Kong, China

## Abstract

Many real-world applications, including adaptive radar scanning and smart stealth, require reconfigurable multifunctional devices to simultaneously manipulate multiple degrees of freedom of electromagnetic (EM) waves in an on-demand manner. Recently, kirigami technique, affording versatile and unconventional structural transformation, has been introduced to endow metamaterials with the capability of controlling EM waves in a reconfigurable manner. Here, we report for a kirigami-inspired sparse meta-architecture, with structural density of 1.5% in terms of the occupation space, for adaptive invisibility based on independent operations of frequency, bandwidth, and amplitude. Based on the general principle of dipolar management via structural reconstruction of kirigami-inspired meta-architectures, we demonstrate reconfigurable invisibility management with abundant EM functions and a wide tuning range using three enantiomers (A, B, and C) of different geometries characterized by the folding angle *β*. Our strategy circumvents issues of limited abilities, narrow tuning range, extreme condition, and high cost raised by available reconfigurable metamaterials, providing a new avenue toward multifunctional smart devices.

## 1. Introduction

Achieving reconfigurable scattering or invisibility [1] adaptive to environmental variation over a large tuning range is of great importance for a smart radar system [2]. As of today, extensive investigations have been carried out to achieve functional reconfigurable devices based on electrical tuning by using varactor [3–9], PIN diode [10–12], Schottky diode [13], photodiode [14], micro-electro-mechanical systems (MEMS) [15], and even nano-electro-mechanical systems (NEMS) [16]. Nevertheless, these schemes involve an external knob and thus typically suffer from additional losses, complex systems, and limited tuning ranges, specifically when operating at microwave frequencies with long wavelengths. Moreover, the configurable and independent control of multiple degrees of freedom incorporating frequency and amplitude is difficult and was rarely realized. Only recently, the available scheme was reported via synergizing dual tuning mechanisms of using varactor and graphene, which however significantly complicates the design [9]. In addition, the band for reflection amplitude modulation is extremely narrow. Other tunable techniques by using transparent conductive oxide [17], liquid crystals [18–22], phase change materials (PCMs) [23] like vanadium dioxide [24–27], and germanium-antimony-tellurium [28] and hydrogenation [29, 30] have also been reported, which are good candidates for light wave manipulation. Nevertheless, they typically require extreme conditions of applied biases, temperature variances, or high-intensity ultrafast pulsed laser beams.

Mechanical tuning offers an effective alternative to switch the electromagnetic (EM) properties of metadevices by structural configurations of constitutive meta-atoms. This is especially true for the origami and kirigami architectures [31–37], which offer unprecedented degrees of freedom and capability in reshaping light without operation band limitation [38–44], including a notable achievement of reconfigurable chirality [38–40]; however, the placement of metapixel is manually configured according to the fold lines of specific origami/kirigami pattern, requiring demanding manufacturing technology for repeatable operations. Such an issue was alleviated to some extent by attaching large numbers of thin metaplates to a three-dimensional (3D) printing platform with predicted shapes [41, 42], which, nevertheless, increases the fabrication cost especially when various continuously folding states and customized functionalities are demanded. Most importantly, the functionalities realized so far are still monotonous, limiting their usage for practical applications.

**Figure 1:**
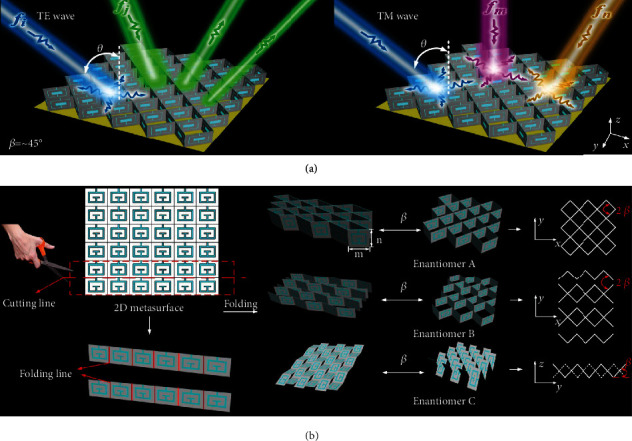
Schematic illustration of reconfigurable invisible metadevices. (a) Conceptual versatile multifunctions and (b) construction of our devised reconfigurable metadevices based on kirigami-metagrouped ITO strategy. A single metamaterial sheet enables independent control of frequency, bandwidth, and amplitude in a simple and continuous manner under external mechanical stimuli. Here, three deformations of enantiomers A, B, and C were realized with numerous various states (*β*), and light with different colors represents different operation wavelengths (*f*_*i*_, *f*_*j*_, *f*_*m*_, and *f*_*n*_) of absorption. Enantiomers A and B are realized by fixing one sheet and arranging the other by a mirror operation along the *x*-axis with and without a separation (*d* = 0 and 2 mm) to the former fixed sheet. The meta-atom lengths (*a*_*x*_, *a*_*y*_, and *a*_*z*_) of enantiomers A and B along *x*-axis, *y*-axis, and *z*-axis are 2∗*m*∗cos*β*, 2∗*m*∗sin*β*, *n* and 2∗*m*∗cos*β*, 2∗*m*∗sin*β* + *d*, *n* according to folding principle. Enantiomer C is constructed by rotating both metasheets along the *x*-axis to form convex mountain and concave valley and arrange them shoulder-to-shoulder by a staggered position of *m*∗cos*β*. The meta-atom lengths of it along *x*-axis, *y*-axis, and *z-*axis are 2∗*n*, 2∗*m*∗cos*β*, and *m*∗sin*β*.

In light of above challenges, we here propose a kirigami-inspired transformable meta-architecture for independent control of the degrees of freedoms including frequency, bandwidth, and amplitude in an on-demand and continuous manner. Based on this strategy, we devise reconfigurable invisible metadevices capable of abundant EM wave manipulations: broadband angle-insensitive absorption at *f*_*i*_ under both transverse electric (TE) and transverse magnetic (TM) waves, TE-dependent amplitude-agile absorption at *f*_*j*_, and TM-dependent frequency-agile absorption, as shown in Figure 1(a). Such a rich manipulation of light is realized by mechanically transforming collectives of identical 2D metapixels to 3D metapixels with a controlled folding angle *β* along the folding line; see Figure 1(b). Specifically, each metapixel is made of two transparent indium tin oxide (ITO) concentric split-ring resonators (SRRs) that induce the dipole. In implementation of above reconfigurable metadevices, we first fabricated a planar flexible metaplate composed of a large number of abundant metapixels and then trim it into various metasheets along cutting line orientated along the *x*-axis. Subsequently, the metasheets are paired up and arranged in different configurations by fixing one sheet while appropriately altering the other in free space through operations of mirror, translation, and rotation. Finally, the metasheet pairs are periodically distributed along the *y*-axis to achieve different transformable metadevices. Therein, different magnetic momentums can be spatially stimulated and reorganized according to the prescribed *β*. For verification, three transformable metadevices are constructed by inserting aforementioned metasheets of different fold angles into etched zig-zag slits of a rigid foam; see Materials and Methods. Experimental results agree very well with the FDTD calculations, well validating the predicted reconfigurable multifunctions.

## 2. Results

### 2.1. Concept and Theoretical Design

To understand how our transformable metamaterials mold light scattering, we first present the general design principle via solving the light scattering using a coupled dipole model. Based on our kirigami-inspired schemes, we start from the periodic meta-atom array where each meta-atom is composed of four electric dipoles, closely placed but with different orientations. The EM response of such a dipole array is described by the well-known set of coupled dipole equations [45, 46]:

(1) $$\begin{array}{c} p_{j}\left(\omega \right)=\alpha \left(\omega \right){\hat{e}}_{j}{\hat{e}}_{j}\left[E_{\text{inc}}\left(r_{j}\right)+\frac{\omega^{2}}{c^{2}}\underset{i\neq j}{\sum }G_{0}\left(\omega ,r_{j},r_{i}\right)p_{i}\left(\omega \right)\right], \end{array}$$

where *c* is the speed of light in vacuum, *E*_inc_(*r*_*j*_) is the external incident field, *α*(*ω*) is the effective polarizability, and ${\hat{e}}_{j}$ is the unit vector of *j*th dipole's orientation. *G*_0_ (*ω*, *r*_*j*_, *r*_*i*_) is the free-space dyadic Green's function describing the propagation of field emitting from the *i*th dipole to *j*th dipole:

(2) $$\begin{array}{c} G_{0}\left(\omega ,r_{j},r_{i}\right)=\frac{\exp\left(ikr\right)}{4\pi r}\left[\left(\frac{i}{kr}-\frac{1}{k^{2}r^{2}}+1\right)I+\left(-\frac{3i}{kr}+\frac{3}{k^{2}r^{2}}-1\right)\hat{r}\hat{r}\right], \end{array}$$

where *k* = *ω*/*c* is the free-space wave number, *r* = |*r*| = |*r*_*j*_ − *r*_*i*_| is the distance between two dipoles, and $\hat{r}$ is the unit vector of *r*.

For the incidence of a plane wave with in-plane momentum *k*_||_(*k*_*x*_, *k*_*y*_), by applying the Bloch theorem, the dipole moment within meta-atom can be expressed as *p*_*m*_(*ω*)exp(*ik*_*x*_*x* + *ik*_*y*_*y*) with *m* = *a*, *b*, *c*, *d* according to the orientation of dipole. From these equations, each dipole moment can be numerically obtained and then the total electric field amplitude at position *l* is given by the following:

(3) $$\begin{array}{c} E\left(l\right)=E_{\text{inc}}\left(l\right)+\frac{\omega^{2}}{c^{2}}\underset{i}{\sum }G_{0}\left(\omega ,l,r_{i}\right)p_{i}\left(\omega \right). \end{array}$$

In addition, the collective response between magnetic dipoles shares the same operation mechanism as that of electric dipoles. Specially, the interaction between the two dipoles (electric or magnetic one) is explained in detail in Supplementary Figure S1. From those formulas, one can easily see that the distance (associated with the element periods in this work) and the orientations between dipoles are two significant mechanisms that dominate the far-field response of the system, which are to be exploited in our transformable metamaterial system for reconfigurable scattering, as discussed below.

**Figure 2:**
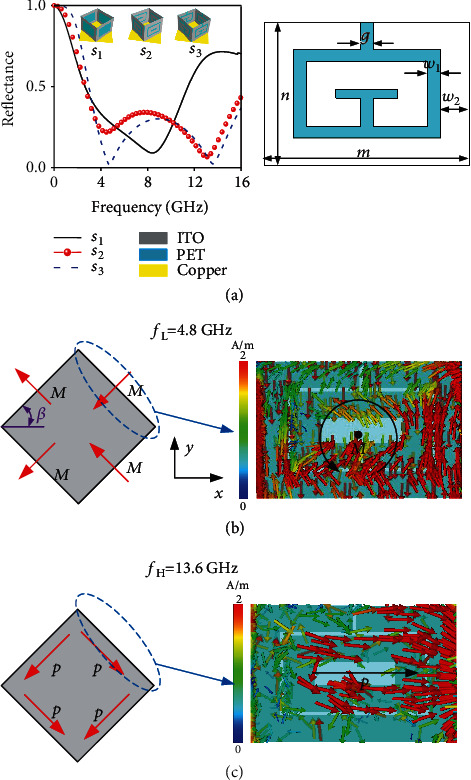
FDTD characterization of the basic meta-atom A (*β* = 45°). (a) FDTD calculated EM reflection spectrum (left panel) of various meta-atoms with the topology and parametric illustration shown in the inset and right panel, respectively. Here, detailed geometric parameters are designed as *m* = 16 mm, *n* = 12 mm, *w*_1_ = 0.3 mm, *w*_2_ = 2.3 mm, and *g* = 0.3 mm. FDTD calculated surface current distributions at (b) low resonance of *f*_L_ = 4.8 GHz and (c) high resonance of *f*_H_ = 13.6 GHz, respectively.

According to aforementioned coupled dipolar model, we now proceed to design meta-atoms exhibiting desired EM scattering properties by tuning $\hat{r}$ of the constituent dipoles through kirigami configuration. As shown in Figure 1(b), each building block is a quad vertical facet, sandwiched by dual-layer concentric ITO split-ring resonators. Here, the square resistance of ITO is selected as *R* = 100 ohm/sq for a tradeoff of absorption and electric conductivity *σ* (*σ* = *L*/*RS*), while the center polyethylene glycol terephthalate (PET) layer has the thickness of 0.1 mm and dielectric constant 3 + 0.003*i*. Such dual-layer architecture supports enhanced resonant effect and will shift magnetic dipolar responses to lower frequency while electric dipolar responses to higher frequency, as described in Figure 2(a). Each meta-atom consists of four vertical facet building blocks, and the way how the building blocks are assembled into a meta-atom changes the overall scattering properties of meta-atoms.

The first exemplary meta-atom, dubbed meta-atom A as depicted in Figure 1(b) and Figure 2(a), is composed of four vertical facets forming a parallelogram on a backed metallic ground. Within each unit cell, four dipoles, either perpendicular or parallel to four sides of the parallelogram of meta-atom A, contribute to the scattering. Here, the parallelogram is realized by folding each two individual PET sheets with an acute angle *β* along the folding line of each pixel and grouping them as a neighbor. Two other enantiomers (meta-atoms B and C) are readily realized by appropriately transforming meta-atom A based on kirigami-inspired strategy. Specifically, meta-atom B is constructed by further separating the two sheets of meta-atom A with a distance *d* while meta-atom C is realized by rotating meta-atom B along the *x*-axis and arranging them shoulder-to-shoulder without separation. In the following theoretical analysis, we take the EM response of meta-atom A as an example, while the response of enantiomers B and C can be analyzed in a similar manner. From the FDTD calculated current distributions at two dips shown in Figure 2(b), the surface current encircles the longitudinal axial of each of the SRRs at *f*_L_ = 4.75 GHz, forming a loop current and resembling the effect of a magnetic dipole with longitudinal coupling. As a result, we achieve four magnetic dipoles perpendicular to the facets. Alternatively, at high frequency *f*_H_ = 13.6 GHz, the surface current in each facet moves along the same direction, forming four electric dipoles parallel to the facets and with transverse coupling (Figure 2(c)).

Thus, we can then treat SRRs at *f*_L_ and *f*_H_ as coupled dipole array composed of magnetic and electric dipoles with longitudinal and transverse coupling. A synergistic cascading of these two resonant modes would contribute a broadband absorption *A* (*A* = 1 − *R*^2^ and *R* represents the reflection coefficient). The mechanical tunablity of *f*_H_ and *f*_L_ in different manners using our kirigami-inspired transformable architecture can be attributed to the periods and relative orientations as functions of *β*. At low frequency *f*_L_, the unit vectors of the four dipoles in this specific case are given by the following:

(4a) $$\begin{array}{c} {\overset{⟶}{e}}_{a}={\overset{⟶}{e}}_{b}=-\cos\left(\beta \right){\overset{⟶}{e}}_{x}+\sin\left(\beta \right){\overset{⟶}{e}}_{y}, \end{array}$$

(4b) $$\begin{array}{c} {\overset{⟶}{e}}_{c}={\overset{⟶}{e}}_{d}=-\cos\left(\beta \right){\overset{⟶}{e}}_{x}-\sin\left(\beta \right){\overset{⟶}{e}}_{y}. \end{array}$$

The periods of meta-atom array are represented as follows:

(5a) $$\begin{array}{c} a_{x}=2m\cos\left(\beta \right), \end{array}$$

(5b) $$\begin{array}{c} a_{y}=2m\sin\left(\beta \right). \end{array}$$

The high frequency *f*_H_ can be deduced in the same manner. A direct comparison of EM response among current SRRs meta-atom and its counterpart made of single closed ITO ring (*S*_1_) and dual closed ITO rings (*S*_2_) without split indicates that our strategy exhibits the best dipole excitation and thus strongest resonant intensity. The polarization-immune EM response of our SRRs ITO meta-atom is explained in detail in Supplementary Figure S2a. Importantly, the dual-layer back-to-back ITO SRRs reduce significantly the magnetic resonance by 37.9% and enhance the electric resonant intensity considerably (electric coupling of two electric dipoles) with respect to the single-layer ITO SRRs; see Supplementary Figures S2b–S2f. The red shift of magnetic resonance arises from the enhanced longitudinal coupling between two magnetic dipoles along the same direction. Here, the optimum geometrical parameters of each enantiomer are finalized such that the resulting metadevices manifest strong resonance and broadband invisibility at *β* = 45°. For this purpose, we obtain a database of reflected spectra of all possible geometries based on massive parametric analyses; see Supplementary Figure S3.

Thus, the dipole coupled mode model can be employed to explain all manifested EM behaviors, well validating the rationality of the theoretical model which will be utilized as a guideline for mechanical tunability. The key physics lies in that the relative position between the dipoles $\hat{r}$ is a strict function of *β* following the folding principle detailed below in three scenarios, and thus, the working frequency can be continuously modulated by tuning *β* in terms of external force. The absorption of EM wave is dependent on the distance of induced dipoles in the metadevices. To achieve reflection amplitude control, we can change it by changing *β*. In the following, we devise several mechanically tunable functional devices for demonstration and possible applications.

### 2.2. Frequency- and Amplitude-Agile Invisibility

**Figure 3:**
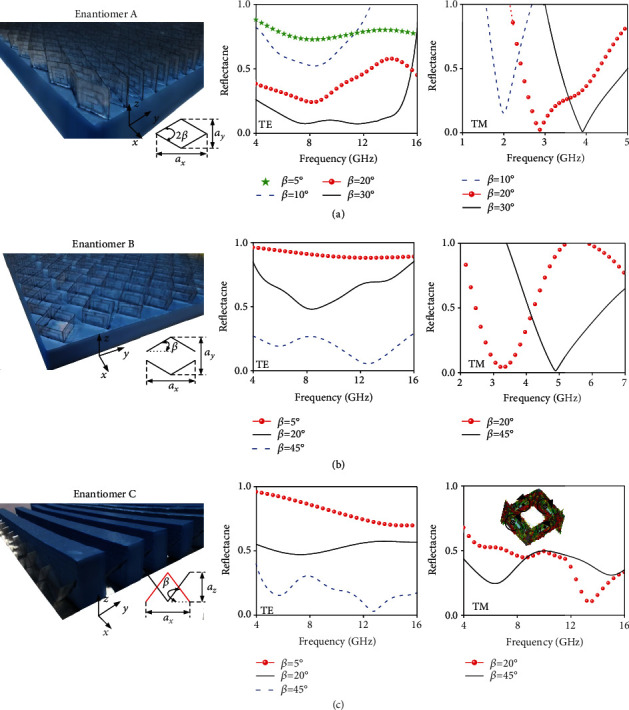
Layout and experimental characterizations of three metadevices of (a) enantiomer A, (b) enantiomer B, and (c) enantiomer C at different folding angles *β* by fixing metamaterial sheets along artificially etched slits on the rigid foam. The left panel shows the photograph of the fabricated sample, while the middle and right panels illustrate the amplitude- and frequency-agile multiability under TE and TM polarizations, respectively. The periodicities of enantiomer A along the *x*-axis and *y*-axis are given as *a*_*x*_ = 2*m*cos*β* and *a*_*y*_ = 2*m*sin*β*. The reason for different *β* of each enantiomer under TE and TM waves is due to limited spectrum of available horn in experiments, especially for the absence of low-frequency horn for small *β* under TM case.

Following the above-established theory, we now demonstrate on-demand configurable spectral responses based on enantiomers A, B, and C. Here, we first show the frequency- and amplitude-agile invisibility management for different *β* under TM (*E*||*x*) and TE (*E*||*y*) wave excitations, respectively. Since the metadevice exhibiting 45° ≤ *β* ≤ 90° is equivalent to that with *β* ≤ 45°, all manipulations are performed within latter range.  Figure 3 shows the layout and experimental results of three metadevices, made of enantiomers A, B, and C, respectively. For those devices, depending on incident polarizations, the amplitude or the peak absorption frequency can be easily modulated by the mechanical reconfigurability according to aforementioned coupled dipolar theory, as indicated by experimental measurements in Figure 3 as well as numerical results in Supplementary Figures S4a–S4c.

Specifically, for polarization-controlled amplitude and frequency responses in enantiomer A (see Figure 3(a)), the reflection rate drops from 75% to 10% when *β* varies within 5°~30° for TE wave while the absorption band, identified from the reflection dips, exhibits a blue shift from 2 to 3.9 GHz when *β* changes from 10° to 30° for TM wave. As a consequence, considerable tuning range is achieved with a fractional bandwidth of Δ*f*/*f*_0_ = 64.4%. The underlying physics can be understood from the established dipole coupling theory. Since the distance (*m*) between the two face-to-face SRRs is constant as *β* varies, the induced magnetic or electric coupling in both TE and TM cases is trivial and is not discussed here. However, this is not the case for the coupling between the two adjacent SRRs. For TE wave, the distance between magnetic dipoles formed between adjacent SRRs along the *x*-axis decreases from *m* to *m*cos*β* while that between formed electric dipoles along the *y*-axis increases from 0 to *m*sin*β*. For TM wave, the distance between magnetic dipoles formed between adjacent SRRs along the *y*-axis increases from 0 to *m*sin*β* while that between formed electric dipoles along the *x*-axis decreases from *m* to *m*cos*β*. As is known, the trigonometric functions exhibit much sharp variation in 0 ~ *m*sin(*β*) region than that in *m* ~ *m*cos(*β*) region. Therefore, the transverse electric coupling changes sharply as *β* and virtually dominates the EM modulation of the metastructure in TE case while the longitudinal magnetic coupling dominates the variation of EM response in TM case. According to the dipole coupled criterion shown in Supplementary Figure S1, both *f*_L_ and *f*_H_ should move toward lower and higher frequencies in TE and TM cases, respectively. However, *f*_L_ shift much slower than *f*_H_ in TE case, and thus, a wider and deeper absorption is expected for a larger *β* in terms of two approaching modes. Nevertheless, the magnetic mode in TM case (largest $\overset{⟶}{n}\cdot \overset{⟶}{H}$ when *β* = 0°, $\overset{⟶}{n}$ is the normal of SRR plane) is with much lower resonant frequency than that in TE case for small *β*, accounting for the separated pure magnetic absorption mode. For further verification, we performed more FDTD calculations on enantiomer A by scattering polarization along the rhombus side to eliminate the complex effects of two vector components. As shown in Supplementary Figure S5, similar results of amplitude control under TE wave and blue shift frequency control under TM wave are expected.

Above analysis according to dipolar interactions can be directly extended to enantiomers B and C. Compared to enantiomer A, the dipole distance of enantiomer B changes from 0 to *m*sin*β* + *d* along the *y*-axis, and the periodic change trend along the *x-*axis is the same as that of enantiomer A. Therefore, similar behavior will be expected for enantiomer B except for more degrees of freedom for tunability and sharper shift of invisibility band due to more rapid changes of interaction strength between face-to-face dipoles. Specifically, the reflection intensity drops from 90% (*β* = 5°) to 10% (*β* = 45°) under TE wave excitation, while under TM wave, the invisibility band shifts from 3.2 to 4.9 GHz as *β* increases from 20° to 45°, corresponding to a fractional bandwidth of 42%; see Figure 3(b).

However, the working mechanism of enantiomer C under TM and TE wave excitations is quite different from that of enantiomers A and B due to the inclined configuration of meta-atoms with completely different periodicities *a*_*y*_ = 2*m*cos*β* and *a*_*z*_ = *m*sin*β* along the *y*-axis and *z*-axis; see Figure 3(c). As indicated by the surface current distributions at resonance of TM wave in the right panel of Figure 3(c), a magnetic dipole is formed on each surface. Two magnetic dipoles of two adjacent surfaces along the *y*-axis are longitudinally coupled in the same direction. Since the distance between two adjacent dipoles decreases as *β* increases, the resulting interaction increases and thus makes the system stable and reduces the systematic restoring force, which enables the resonance-induced invisibility to lower band. Therefore, we expect a red shift of invisibility band from 13.3 to 5.8 GHz as *β* increases from 20° to 45°, offering the largest tunable range with fractional bandwidth of 78.5%. For TE wave, the reflection rate reduces from 70% to 5% as *β* increases from 5° to 45° due to the enlarged absorption area in terms of increased *a*_*z*_. This is especially true for the superior absorption at *β* = 45°.

### 2.3. Broadband Angle-Insensitive Invisibility

**Figure 4:**
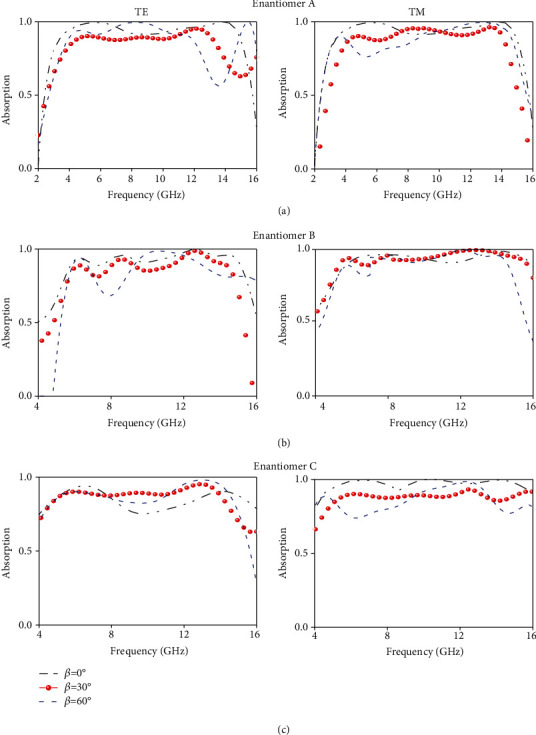
Experimentally measured absorption of proposed transformable metadevices transformed among three states of (a) enantiomer A, (b) enantiomer B, and (c) enantiomer C when incident angle (*θ*) varying from 0 to 60° under TE (left panel) and TM (right panel) polarizations. Note that the absorption is related to physical volume of the transformable metadevices which reaches their maximum when the folding angle is *β* = 45° according to *v* = *a*_*x*_*a*_*y*_*a*_*z*_, where *v*_*A*_ = 6144sin2*β*, *v*_*B*_ = 6114sin2*β* + 768cos*β*, and *v*_*c*_ = 3072sin2*β*.

We now show that broadband wide-angle absorption is supported in our system at the specific folding angle, especially at the critical angle of *β* = 45° according to the dipole interaction pictures illustrated in Section 2. This is a quite interesting case for enantiomer A, because, at this *β*, *a*_*x*_ = *a*_*y*_, and the distances between electric dipoles along electric field and magnetic dipoles along magnetic field reach to their minimum or maximum. Specifically, both the magnetic mode with longitudinal coupling at *f*_L_ and the electric mode with transverse coupling at *f*_H_ exhibit a red shift (blue shift) of invisibility band as *β* increases to 45° in TE (TM) case. However, as discussed previously, *f*_L_ changes much slower than *f*_H_ in TE case while inversely much faster in TM case. As a consequence, in both cases, two modes approach to each other and manifest a broadband absorption at *β* = 45°. Moreover, the volume of the metadevices also reaches its extreme, accounting for the best absorption. Besides, the meta-atom exhibits higher fourfold rotational symmetry, indicating polarization-independent EM responses. Quantitatively, the absorption reaches above 90% within 3.7~15.4 GHz for both TE and TM waves of normal incidence, corresponding to an absolute and fractional bandwidth of 11.7 GHz and 122.5%; see Figure 4(a). Specifically, at the incident angles (*θ*) of *θ* = 60°, the absorption reaches even more than 80% within 3.8~12 GHz for TE wave while that is observed more than 75% within 3~15.5 GHz under TM wave.

Since enantiomers B and C share the physical principle of dipole interaction and their volume also reaches the extreme value, both exhibit broadband absorption at *β* = 45° but with a different broadband operation spectrum. By transforming metadevices among enantiomers A, B, and C, flexible invisibility with reconfigurable spectrum can be engineered and integrated using only one set of metamaterials of different transformations. To showcase this idea, we also numerically and experimentally characterize enantiomers B and C at *β* = 45°. As shown in Figure 4(b), the absorption reaches more than 80% within 6~15.8 GHz at both TE and TM waves of normal incidence, corresponding to an absolute and fractional bandwidth of 9.8 GHz and 90.7%. As shown in Figure 4(c), the absorption reaches more than 80% within 4.3~15.6 GHz at both TE and TM waves, and thus, the absolute and fractional bandwidth is achieved as 11.3 GHz and 113.6%. Moreover, the absorption still approaches more than 70% even up to *θ* = 60° at both TE and TM waves for enantiomers B and C. In all cases of three metadevices, experimental results agree very well with FDTD calculations; see Supplementary Figures S6a–S6c. Slight deviations between the numerical and experimental results are likely induced by the inaccuracy of sample's angles during the sample fabrication and partially by the imperfect nonplanar incoming wavefront in measurements. This is especially true for the cases of small angles under TE wave incidence, where more parallelogram metasheets are necessary and thus induce larger tolerances, accounting for the relatively large deviations in the case of *β* = 10° observed in Figure 3(a) and Figure S4a. Our results show broadband angle-insensitive invisibility configurable via our mechanically transformable metamaterials, with great potentials in stealth applications.

### 2.4. Advantages of Proposed Kirigami-Inspired Architectures

**Figure 5:**
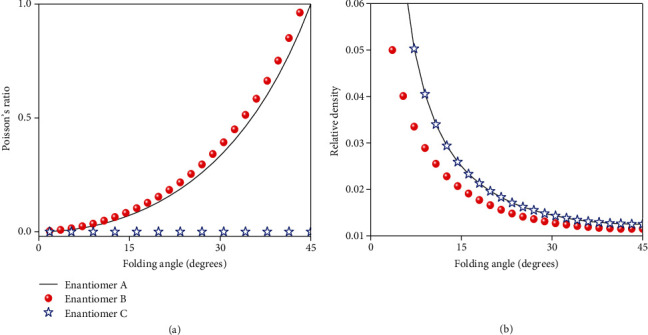
Theoretically calculated (a) Poisson's ratio and (b) relative density as a function of different *β* of the transformable metadevices at three enantiomer states of A, B, and C.

Lastly, we discuss the relations and advantages of our three metadevices, especially from the perspective of the Poisson's ratio and relative density. The Poisson's ratio refers to the scale of absolute value of transverse contraction strain to longitudinal tensile strain in the direction of stretching force along the *y*-axis. Here, it is numerically investigated and defined as *v* = −(*dl*/*l*)/(*dw*/*w*), where *l* and *w* are the length and width of meta-atom, respectively. Specifically, *v* is calculated as *v*_1_ = tan^2^*β* and *v*_2_ = (1 + 1)/(*m*sin*β*) for enantiomers A and B, respectively. However, it is infinite for enantiomer C since there is no change of meta-atom along the *x*-axis (*dw* = 0).  Figure 5(a) shows the numerically calculated *v* as a function of *β*. As is clearly inspected, the Poisson's ratio enhances as *β* increases. As a consequence, the lateral deformation of the metadevice becomes large, accounting for a larger elastic stress. Moreover, the Poisson's ratio of enantiomer B is larger than that of enantiomer A, indicating the elastic stress of enantiomer B is larger than that of enantiomer A.

The relative density *ρ* is also another important feature of our transformable metadevices, which can be substantially reduced by folding. This quantity *ρ* = *t* · (*mn*)/(*HLW*) is calculated as the ratio between the volumes before and after folding, where *m*, *n*, and *t* are the length, width, and thickness of the 2D meta-sheet, respectively, and *L*, *W*, and *H* are the length, width, and height of the 3D meta-atom, respectively. According to folding principle of our transformable metadevices, *ρ* is formulated as *ρ*_1_ = 1/80sin2*β*, *ρ*_2_ = 1/(80sin2*β* + 10cos*β*), and *ρ*_3_ = 1/80sin2*β* for enantiomers A, B, and C.  Figure 5(b) portrays these theoretically calculated *ρ* as a function of *β*. Specifically, the relative density decreases as *β* increases in all cases, while it manifests identical value and variation for enantiomers A and C. The *ρ* of enantiomer B is smaller than that of enantiomer A, indicating the weight of enantiomer B is lighter when constructing metadevice in identical volume at the same *β*. Quite interestingly, the minimum relative density dramatically reduced to less than 1.5% that of the unfolded structure when *β* = 45°. This fascinating characteristic is extremely beneficial to lightweight metadevices in practical applications.

## 3. Discussion

To sum up, we have demonstrated theoretically and experimentally a kirigami-inspired transformable meta-architecture for independent control of freedoms of frequency, bandwidth, and amplitude in an on-demand and continuous manner according to the dipole coupling theory. By transforming among three enantiomers of our reconfigurable metadevice, three functions with adaptable invisibility management were realized. Specially, the experiments show the reflection drops from 75% to 10% and the absorption reaches more than 90% within 3.7~15.4 GHz (a fractional bandwidth of 122.5%) by a continuous tuning of folding angles for enantiomer A. Moreover, Poisson's ratio and relative density show that our metadevice features light in density and small in volume, promising great potentials in satellite applications in outer space where attractive lightweight is the major concern. Therefore, our strategy provides an unprecedented alternative and paves up a solid platform for metadevices with reproducibility and multifunctions.

## 4. Materials and Methods

### 4.1. Numerical Characterizations

All EM wave simulations are performed through the commercial software of CST Microwave Studio 2018. When calculating reflection coefficients of the meta-atom in frequency-domain solver, unit cell boundary conditions were assigned along the *x*-axis and *y*-axis while open boundary conditions were set up along *Z*_max_ direction and electric boundary was adopted at *Z*_min_.

### 4.2. Sample Preparation and Fabrication

**Figure 6:**
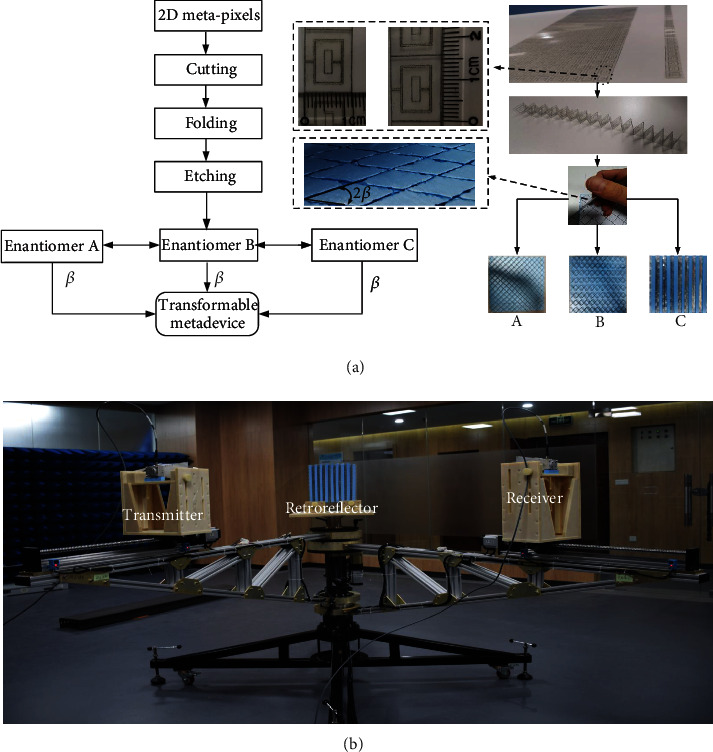
Illustration of the fabrication process (a) and experimental setup (b).

All samples are prepared based on a four-step fabrication process shown in Figure 6(a). First, cut the 2D metasurfaces into metasheets with scissors or cutters, followed by folding the metasheet according to the folding line. The third step is to etch groove slots on the foam board with dielectric constant near unity and then insert the folded metasheets into the slots. In order to ensure the accuracy of the folding angle, we print the accurate angles on the computer, paste it subsequently on the foam board, and then etch it in the accurate angles. The last is pasting the metadevices attached to the foam board on the metal plate. Metadevices with different *β* are similar. We only need to print metasurfaces with different angles and then etch the structure on the foam board. We can disassemble and recycle the metal plate and insert them into another well-etched foam board when measuring different geometries.

### 4.3. Microwave Experiments

All experiments are performed in open areas; see the experimental setup shown in Figure 6(b). Two linear polarization antennas are utilized as receiver and transmitter. The double-ridged horn produced a voltage standing wave ratio less than 2.0 within the frequency of 2~18 GHz is used as the linear polarization antenna. The two antennas are connected to an AV3672B vector network analyzer to transmit and receive the EM wave signals. Through changing the orientation of the two antennas with respect to the fixed retroreflector, a linear polarization wave excitation can be easily realized with several polarization angles of 0°, 30°, and 60°.

## Data Availability

The data used to support the findings of this study are available from the corresponding authors upon request.

## Supplementary Materials

Supplementary Materials Figure S1: energy-level diagram describing the transverse and longitudinal coupling between two dipoles. Figure S2: FDTD calculated reflection spectrum of meta-atoms in different cases to illustrate the evolved design process of enantiomer A. In all cases, the folding angle is *β* = 45°. Here, *ryy* (*rxx*) represents the output copolarized components under excitation of normally incident *y*-polarized (*x*-polarized) planar wave. (a) The finally designed meta-atom A-based dual-layer back-to-back ITO SRRs. (b) Parametric illustration of SRRs. Dual-layer meta-atoms based on (c) single and (d) dual closed ITO rings without split. Single-layer meta-atoms based on ITO SRRs placed in (e) inner and (f) outer faces. Figure S3: parametric analyses for different parameters of meta-atom (enantiomer A, *β* = 45°). In each case, only one parameter is changed while other parameters are fixed. The effect of (a) height *h* = *n*, (b) *m*, (c) *w*_1_, (d) *w*_2_, (e) *g*, and (f) *d* of the structure. Figure S4: numerically calculated reflection spectrum of the metadevice at three different transformable states of (a) enantiomer A, (b) enantiomer B, and (c) enantiomer C. By changing different folding angles *β*, the reflection coefficient of EM waves can be controlled under TE polarization, and the resonant frequency can be adjusted under TM polarization. Figure S5: numerical results of enantiomer A at different folding angles (*β*) when polarization is along (a) rhombus side and (b) its adjacent rhombus side. Figure S6: numerical calculated absorption rate of the metadevice at three different transformable states of (a) enantiomer A, (b) enantiomer B, and (c) enantiomer C when the EM wave is obliquely incident from 0° to 60° at TE (left panel) and TM (right panel) polarization states.
