# Supplementary material for: Adaptable Invisibility Management Using Kirigami-Inspired Transformable Metamaterials
Source: Research (Wash D C). 2021 Sep 10;2021:9806789. doi: 10.34133/2021/9806789 (PMC8449819; doi:10.34133/2021/9806789)
Supplement: Supplementary Materials — Figure S1: energy-level diagram describing the transverse and longitudinal coupling between two dipoles. Figure S2: FDTD calculated reflection spectrum of meta-atoms in different cases to illustrate the evolved design process of enantiomer A. In all cases, the folding angle is β = 45°. Here, ryy (rxx) represents the output copolarized components under excitation of normally incident y-polarized (x-polarized) planar wave. (a) The finally designed meta-atom A-based dual-layer back-to-back ITO SRRs. (b) Parametric illustration of SRRs. Dual-layer meta-atoms based on (c) single and (d) dual closed ITO rings without split. Single-layer meta-atoms based on ITO SRRs placed in (e) inner and (f) outer faces. Figure S3: parametric analyses for different parameters of meta-atom (enantiomer A, β = 45°). In each case, only one parameter is changed while other parameters are fixed. The effect of (a) height h = n, (b) m, (c) w1, (d) w2, (e) g, and (f) d of the structure. Figure S4: numerically calculated reflection spectrum of the metadevice at three different transformable states of (a) enantiomer A, (b) enantiomer B, and (c) enantiomer C. By changing different folding angles β, the reflection coefficient of EM waves can be controlled under TE polarization, and the resonant frequency can be adjusted under TM polarization. Figure S5: numerical results of enantiomer A at different folding angles (β) when polarization is along (a) rhombus side and (b) its adjacent rhombus side. Figure S6: numerical calculated absorption rate of the metadevice at three different transformable states of (a) enantiomer A, (b) enantiomer B, and (c) enantiomer C when the EM wave is obliquely incident from 0° to 60° at TE (left panel) and TM (right panel) polarization states. [file 9806789.f1.docx]

Supplementary Materials

**Adaptable Invisibility Management using Kirigami-Inspired Transformable Metamaterials**

By *He-Xiu Xu^1,2*^, Mingzhao Wang^1†^, Guangwei Hu^3†^, Shaojie Wang^1^, Yanzhao Wang^1^, Chaohui Wang^1^, Yixuan Zeng^3^, Jiafang Li^4,5^, Shuang Zhang^6,7*^*, *Wei Huang*^2^*^*^*

*^1^Air and Missile Defense College, Air Force Engineering University, Xi'an 710051, China*

*^2^Institute of Flexible Electronics, Northwestern Polytechnical University, Xi'an 710072, China*

*^3^Department of Electrical and Computer Engineering, National University of Singapore, Singapore 117583, Singapore*

*^4^Centre for Quantum Physics, Key Laboratory of Advanced Optoelectronic Quantum Architecture and Measurement (MOE), Beijing 100081, China*

*^5^School of Physics, Beijing Institute of Technology, Beijing 100081, China*

*^6^Department of Physics, University of Hong Kong, Hong Kong, China*

*^7^Department of Electronic & Electrical Engineering, University of Hong Kong, Hong Kong, China*

†These authors contribute equally to this work

*Corresponding Authors: He-Xiu Xu, E-mail: [hxxuellen@gmail.com](mailto:hxxuellen@gmail.com); Shuang Zhang, E-mail: [shuzhang@hku.HK](mailto:shuzhang@hku.HK); Wei Huang, E-mail: iamwhuang@nwpu.edu.cn

1. **Derivation for transformable interaction energy V for tunability**

In the main text, we have afforded equations to elucidate the physics for mechanical tunablity of *f*_H_ and *f*_L_ in our specified case. Here, we give more details on how to achieve these equations. Supposing that two dipoles (electric or magnetic one) with moments of ***p*_1_** and ***p*_2_** are separated at a distance ***r***, see left panel of **Fig. S1**. Then the quasi-static interaction energy *V* can be formulated as

             (1).

Here, ***r*** represents the distance between the two dipoles and $\varepsilon_{0}$ is the permittivity in free space. When the two dipoles coupled vertically or horizontally, Eq. (1) can be simplified as $V=\gamma\frac{p_{1}\bullet p_{2}}{4\pi\varepsilon_{0}r^{3}}$, where $\gamma$ is the interaction coefficient and is taken as $\gamma=-2$ and $\gamma=+1$, respectively when dipoles coupled longitudinally and transversely. For a pair of electric dipoles shown in right panel of **Fig. S1**, if they are placed transversely along the same (opposite) direction, the coupling between them will increase (decrease) the restoring force of the system and thus enable the dipoles to operate at high (low) frequency. Besides, The smaller the distance |$\hat{r}$| between two dipoles, the greater the repulsive force and thus at the higher resonance frequency is, and vice versa for larger distance, which is well indicated by $\left(\overrightarrow{p}//\overrightarrow{p}\right)\propto f_{H}$($(\overrightarrow{p}//\overleftarrow{p})\propto f_{L}$). However, situation will be reversed if above paired dipoles were distributed longitudinally in line (oppositely) with each other. Therein, the interaction makes the system more (less) stable and thus facilitates to resonate at low (high) frequencies. The smaller |$\hat{r}$| contributes greater attractive force and thus compels the system to shift toward lower frequency, which is theoretically revealed by $\left(\overrightarrow{p}\perp\overrightarrow{p}\right)\propto f_{L}$($(\overrightarrow{p}\perp\overleftarrow{p})\propto f_{H}$). The coupling between paired magnetic dipoles shares the same operation mechanism as that of electric dipoles. At *f*_L_, the specified *V* for the quad magnetic dipoles displaced on rhomb is derived as

  (2),

whereas that for quad electric dipoles on rhomb can be achieved as

(3).

Equation (2) and (3) can be further simplified as

              (4).

               (5).


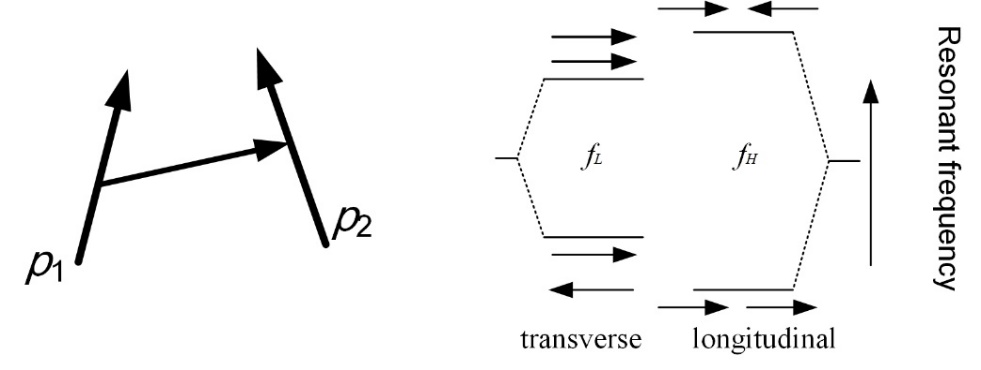


**Figure S1.** Energy-level diagram describing the transverse and longitudinal coupling between two dipoles.

1. **Advantages of our meta-atom design**

In the main text, we have shown the tunable feature (frequency and amplitude management) of our kirigami meta-atom based on the modulated interaction between adjacent dipoles. Here, we further discuss its merits by comprehensive comparisons to other several cases with *β*=45^o^. **Fig. S2 (a)** shows the FDTD calculated co-polarized reflection coefficients of the meta-atom using dual-layer back-to-back ITO SRRs shown in **Fig. S2b** under normally incident *x*/*y*-polarized planar electromagnetic (EM) wave. Almost identical reflection spectrum is expected with reflection below -10 dB in a broad range of 3.5~15.6 GHz at two cases, revealing an elegant broadband polarization-immune absorption. **Fig. S2c** plots the reflection spectrum of the meta-atom only containing a closed ITO ring. As expected, there is only a single magnetic resonant mode with one reflection dip and thus the absorption bandwidth is limited. However, additional mode will be generated at high frequency when an inner closed ITO ring is added. The cascading of these two modes has significantly boosted the bandwidth, see **Fig. S2d**. Nevertheless, the triggered intensity of dipole mode is weak especially at low frequencies and thus the impedance match and -10 dB bandwidth is still limited. Subsequently, we resolved to anisotropic architecture for enhanced dipole resonance. **Figs. S2e** and **S2f** depict the FDTD calculated reflection spectrum of the meta-atom of single-layer ITO split ring resonators (SRRs) placed on inner and outer face. As is shown, the spectrum is observed almost the same in both cases, revealing a strong fundamental magnetic resonance due to the increased capacitance. Although weak resonance at high frequency is not conducive to subsequent frequency and amplitude regulation, it can be easily improved by synergizing dual-layer ITO SRRs, see the sandwiched dual-layer ITO in **Fig. S2a**, where electric and magnetic dipoles are well excited.

**
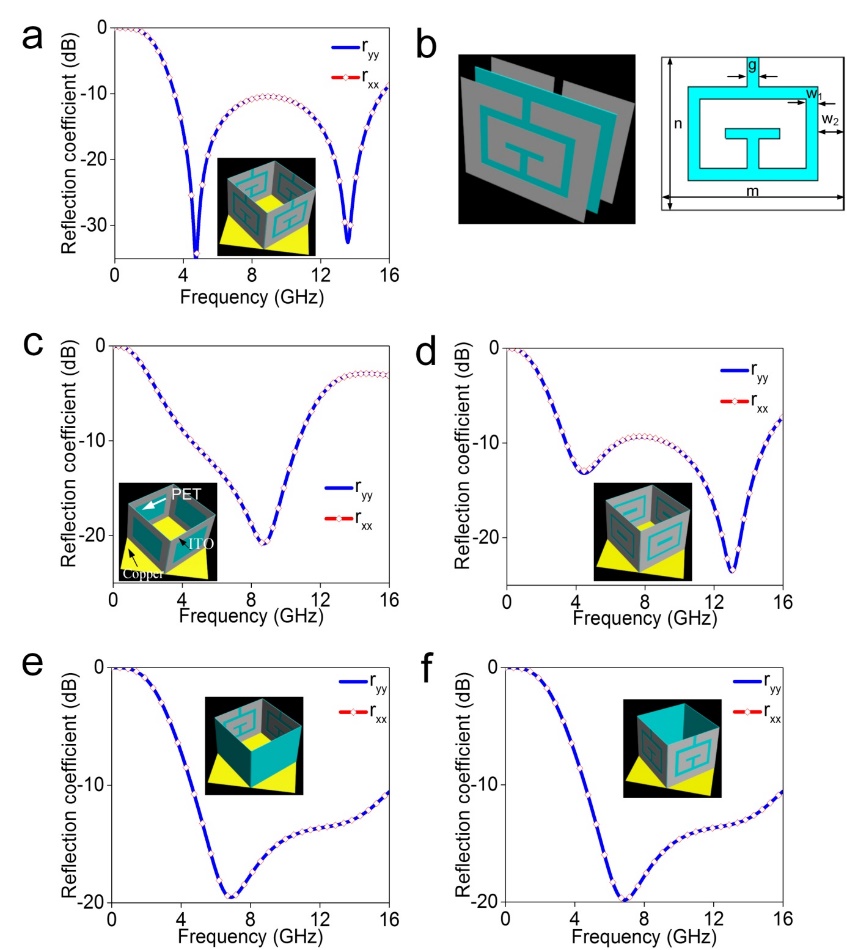
**

**Figure S2.** FDTD calculated reflection spectrum of meta-atoms in different cases to illustrate the evolved design process of enantiomer A. In all cases, the folding angle is *β*=45^o^. Here, *r*_yy_ (*r*_xx_) represents the output co-polarized components under excitation of normally incident y-polarized (x-polarized) planar wave. (a) The finally designed meta-atom A based dual-layer back-to-back ITO SRRs. (b) Parametric illustration of SRRs. Dual-layer meta-atoms based on (c) single and (d) dual closed ITO rings without split. Single-layer meta-atoms based on ITO SRRs placed in (e) inner and (f) outer faces.

1. **Determination of optimum structural parameters**

To ensure an elegant frequency and amplitude tuning range, sufficiently strong resonance and extreme reflection values are necessary. In the following, we conduct parametric analysis to afford a guideline for optimum design of the transformable metamaterials. As shown in **Figs. S3a – S3f**, we analyzed the influence of different parameters on reflection spectrum by changing one parameter while keeping other ones fixed. The key for the altered resonant intensity is the effective area of ITO changed as a function of different parameters. Here, the effective ITO area of enantiomer A is calculated as $\overline{S}=S_{ITO}/p_{x}p_{y}$ ($S_{ITO}$ represents the area of ITO, *a*_x_ and *a*_y_ refer to the periods along *x* and *y* axis). As shown in **Fig. S2a**, the effective area $\overline{S}$ decreases from 86.5% to 76.8% when the height (*h*=*n*) of the meta-atom increases from *n*=12 to *n*=18. Therefore, we expect a greatly degenerated absorption when $\overline{S}$ decreases. The reason for other modulated reflection spectrum as a function of other structural parameters is similar to that of *h* and is thus not detailed for brief of contents*.* Based on these parametric analyses, we determined final parameters of our transformable meta-atom as: *n*=12, *m*=16, *w*_1_=0.3, *w*_2_=2.3, *g*=0.3 and *d*=0.1 mm.


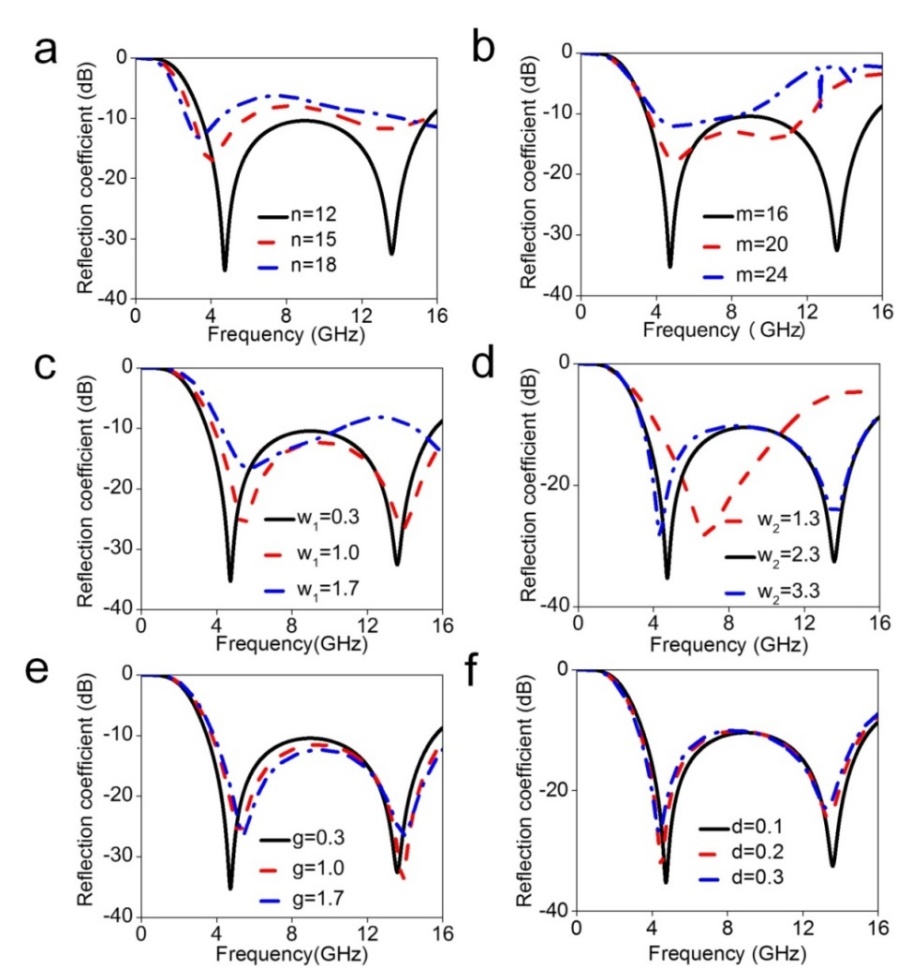


**Figure S3.** Parametric analyses for different parameters of meta-atom (enantiomer A, *β*=45^o^). In each case, only one parameter is changed while other parameters are fixed. The effect of (a) height *h*=*n*, (b) *m*, (c) *w*_1_, (d) *w*_2_, (e) *g*, and (f) *d* of the structure.

1. **Numerical results for frequency- and amplitude-agile invisibility**

In **Fig. 3** of the main text, we have shown the experimental results for adaptable invisible frequency and intensity control by adjusting the folding angle *β* under TE and TM polarizations, respectively. Here, we further afford numerical FDTD calculations for comparisons and better understanding of the resulting distinct functions. As shown in **Fig. S4a**, the simulated results indicate that the enantiomer A manifests a tuning of reflection coefficient from -1 to -22 dB under excitation of transverse electric (TE) polarization when *β* varies from 1.5° to 30°. In contrast, the operation frequency for invisibility of the enantiomer A undergoes sharp frequency shift from 0.7 to 4.7 GHz (a tuning range of 148.1%) when *β* varies from 1.5° to 45° under transverse magnetic (TM) polarization. As shown in Fi**g. S4b**, enantiomer B exhibits a reflection coefficient tuning of -0.5 to -20 dB under TE polarization, while a frequency shift of 1.6 to 4.8 GHz (a tuning range of 100%) under TM polarization when *β* varies within 3°~45°. As shown in **Fig. S4c**, the enantiomer C manifests a continuous reflection variation within -2~-36 dB under TE polarization when *β* varies from 0° to 45° while a frequency shift of 13.5~5.5 GHz (a tuning range of 84.2%) under TM polarization when *β* varies from 20° to 45°. In particular, numerical and experimental results manifest good consistency except for relatively large deviations in the case of *β=*10° under TE polarization. The main reason is that when the angle is small, more parallelogram meta-sheets are necessary which would thus induce larger tolerances (inaccuracy of sample's angles) during the sample fabrication.


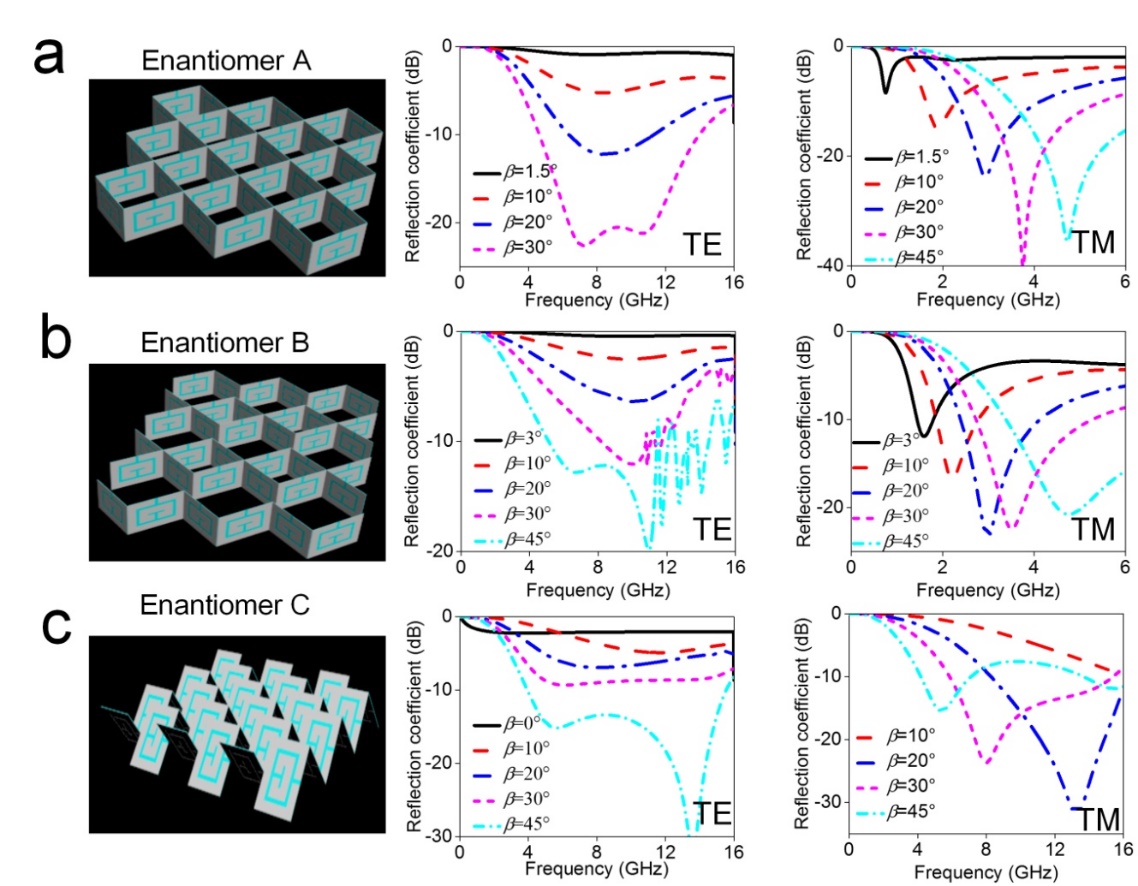


**Figure S4.** Numerically calculated reflection spectrum of the meta-device at three different transformable states of (a) enantiomer A, (b) enantiomer B, and (c) enantiomer C. By changing different folding angles *β*, the reflection coefficient of EM waves can be controlled under TE polarization, and the resonant frequency can be adjusted under TM polarization.

In the main text, we have performed FDTD calculations to demonstrate that the dipole coupling is the origin of the amplitude and frequency control in TE and TM wave case. To further validate that this effect is induced by the dipole coupling rather than the variation of electrical length of the meta-structure (periodicity), here we performed more FDTD calculations on enantiomer A by setting polarizations along the rhombus side and its adjacent rhombus side, respectively, as shown in **Fig S5**. In this case, the effects of two vector components were also eliminated. With the increase of *β*, the reflection for polarization along rhombus side becomes smaller and smaller, while the resonance for polarization along its adjacent rhombus side shifts to high frequency. The results are basically consistent with the results shown above when polarization is along *x*-axis and *y*-axis. Therefore, it can be concluded that the absorption modulation has little to do with the period, but instead mainly caused by the coupling effect of the dipoles. Indeed, the vector of excited magnetic dipole or electric dipole is only along the direction of magnetic or electric field.


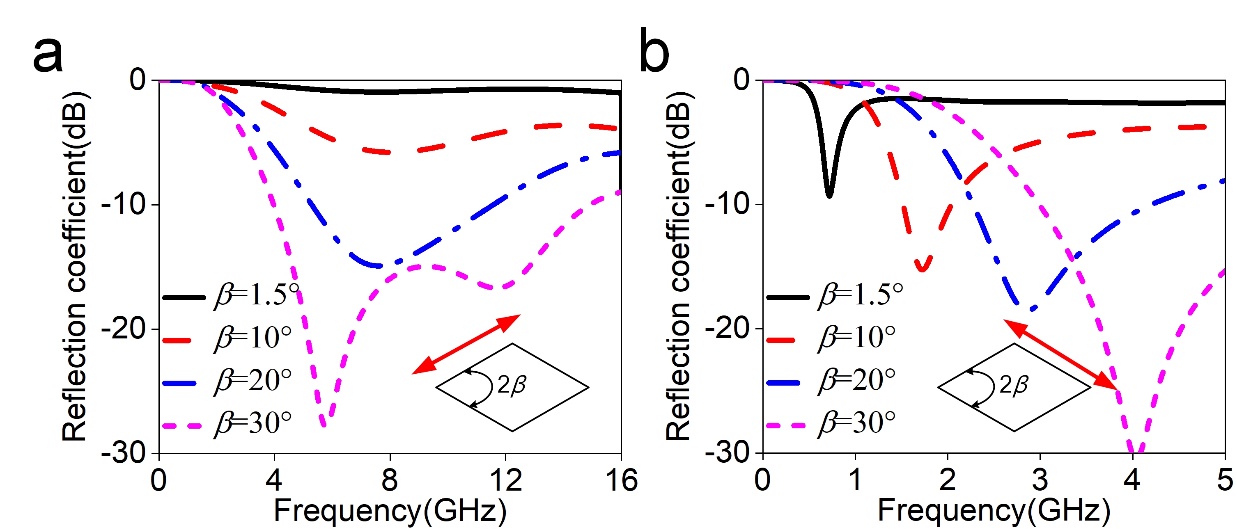


**Figure S5.** Numerical results of enantiomer A at different folding angles (*β*) when polarization is along (a) rhombus side and (b) its adjacent rhombus side.

1. **Numerical results for broadband angle-insensitive invisibility**

In **Fig. 4** of the main text, we have shown the experimental demonstration for broadband angle-insensitive invisibility by fixing *β=*45º and altering oblique incidence angles *θ* within 0~60º in steps of 30º under TE and TM polarizations, respectively. Here, we further afford the FDTD calculated results for verification. As shown in **Fig. S6a**, at the incident angles (*θ*) of 0°, 15°, 30°, 45°, and 60°, the absorption spectrum is very stable for enantiomer A. The absorption rate at both TE and TM wave of normal incidence reaches more than 90% within 3.5 to 15.6 GHz, corresponding to an absolute bandwidth of 12.1 GHz and a relative bandwidth of 126.7%. Up to *θ*=60°, the absorption rate at TM polarization is still more than 75% in the range of 3.4~14.3 GHz while that at TE polarization is more than 90% within 3.5~14 GHz. As shown in **Fig. S6b,** the absorption rate of EM wave reaches more than 80% at 6~16 GHz at both TE and TM wave of normal incidence, corresponding to an absolute bandwidth of 10 GHz and a relative bandwidth of 90.9% for enantiomer B. Specifically, up to *θ*=60°, the absorption rate of TE polarization reaches more than 80% in the range of 4.5~12 GHz, the absorption rate of TM polarization can reach more than 80% in the range of 3.5~13.5 GHz. For enantiomer C as shown in **Fig. S6c,** the absorption rate of TE polarization reaches more than 90% in the range of 4~15.6 GHz at both TE and TM wave of normal incidence, corresponding to an absolute bandwidth of 11.6 GHz and a relative bandwidth of 118.4%. Specifically, up to *θ*=60°, the absorption rate of TE polarization reaches more than 80% in the range of 4~12 GHz, the absorption rate of TM polarization reaches more than 80% in the range of 3.3~12.5 GHz.


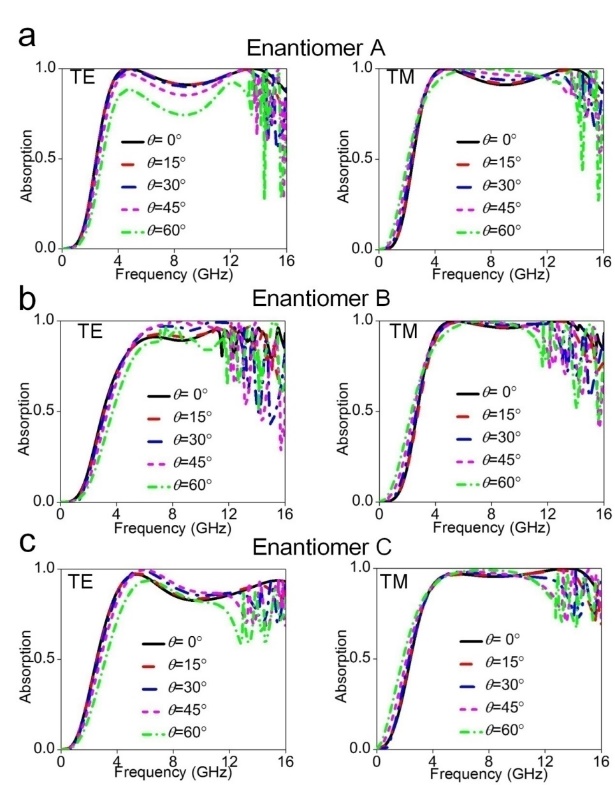


**Figure S6.** Numerical calculated absorption rate of the meta-device at three different transformable states of (a) enantiomer A, (b) enantiomer B, and (c) enantiomer C when the EM wave is obliquely incident from 0° to 60° at TE (left panel) and TM (right panel) polarization states.
